# Association of Insurance Mix and Diagnostic Coding Practices in New York State Hospitals

**DOI:** 10.1001/jamahealthforum.2022.2919

**Published:** 2022-09-02

**Authors:** Kacie L. Dragan, Sunita M. Desai, John Billings, Sherry A. Glied

**Affiliations:** 1 New York University’s Robert F. Wagner Graduate School of Public Service, New York, New York; 2 Interfaculty Initiative in Health Policy, Graduate School of Arts and Sciences, Harvard University, Cambridge, Massachusetts; 3 Department of Population Health, New York University School of Medicine, New York, New York

## Abstract

**Importance:**

Given higher reimbursement rates, hospitals primarily serving privately insured patients may invest more in intensive coding than hospitals serving publicly insured patients. This may lead these hospitals to code more diagnoses for all patients.

**Objective:**

To estimate whether, for the same Medicaid enrollee with multiple hospitalizations, a hospital’s share of privately insured patients is associated with the number of diagnoses on claims.

**Design, Setting, and Participants:**

This cross-sectional study used patient-level fixed effects regression models on inpatient Medicaid claims from Medicaid enrollees with at least 2 admissions in at least 2 different hospitals in New York State between 2010 and 2017. Analyses were conducted from 2019 to 2021.

**Exposures:**

The annual share of privately insured patients at the admitting hospital.

**Main Outcomes and Measures:**

Number of diagnostic codes per admission. Probability of diagnoses being from a list of conditions shown to be intensely coded in response to payment incentives.

**Results:**

This analysis included 1 614 630 hospitalizations for Medicaid-insured patients (mean [SD] age, 48.2 [20.1] years; 829 684 [51.4%] women and 784 946 [48.6%] men). Overall, 74 998 were Asian (4.6%), 462 259 Black (28.6%), 375 591 Hispanic (23.3%), 486 313 White (30.1%), 128 896 unknown (8.0%), and 86 573 other (5.4%). When the same patient was seen in a hospital with a higher share of privately insured patients, more diagnoses were recorded (0.03 diagnoses per percentage point [pp] increase in share of privately insured; 95% CI, 0.02-0.05; *P* < .001). Patients discharged from hospitals in the bottom quartile of privately insured patient share received 1.37 more diagnoses when they were subsequently discharged from hospitals in the top quartile, relative to patients whose admissions were both in the bottom quartile (95% CI, 1.21-1.53; *P* < .001). Those going from hospitals in the top quartile to the bottom had 1.67 fewer diagnoses (95% CI, −1.84 to −1.50; *P* < .001). Diagnoses in hospitals with a higher private payer share were more likely to be for conditions sensitive to payment incentives (0.08 pp increase for each pp increase in private share; 95% CI, 0.06-0.10; *P* < .001). These findings were replicated in 2016 to 2017 data.

**Conclusions and Relevance:**

In this cross-sectional study of Medicaid enrollees, admission to a hospital with a higher private payer share was associated with more diagnoses on Medicaid claims. This suggests payment policy may drive differential investments in infrastructure to document diagnoses. This may create a feedback loop that exacerbates resource inequity.

## Key Points

**Question:**

What is the association between a hospital’s share of privately insured patients and diagnostic coding practices?

**Findings:**

Analyses of 1 614 630 New York Medicaid admissions indicate that for a given patient with multiple hospitalizations, the number of diagnoses on a claim was positively associated with the hospital’s share of privately insured patients. Patients discharged from hospitals in the bottom quartile by share of privately insured patients were coded with 1 to 2 additional diagnoses when they were subsequently discharged from hospitals in the top quartile.

**Meaning:**

Payer mix is associated with diagnostic coding patterns, suggesting that payment policy may be influencing investments in administrative infrastructure.

## Teaser

This cross-sectional study examines the association between a hospital’s share of privately insured patients and diagnostic coding practices.

## Introduction

Administrative infrastructure, such as coding software, is marketed in part as helping hospitals maximize reimbursement.^1,2^ Because hospital reimbursement rates are much higher on average for privately insured patients than for those insured under Medicare or Medicaid,^3^ the stakes for investing in such infrastructure and being thorough in coding diagnoses and procedures may be higher for hospitals serving a larger number of private patients. Most payers use diagnosis- and severity-based payment methodologies,^4,5^ but the promise of higher prices for the same set of diagnoses from private payers may increase the incentive to code intensively. Hospitals serving high shares of privately insured patients also have more resources to purchase these systems and to hire and train administrative teams who can use them.^6^

Prior evidence suggests that higher prices or other features of payment systems incentivize hospitals to increase diagnostic coding. Increases in prices for selected Medicare admissions have been associated with increases in coding into these diagnoses.^7^ Likewise, conversion of hospitals from not-for-profit to for-profit status has been associated with increases in coding intensity.^8^ Literature in Medicare has found that the number of diagnoses on a patient’s record increases when they move to an area with higher-intensity coding behavior, reflecting local administrative coding practices rather than the true underlying health of patients.^9^ Although prior evidence has largely come from Medicare due to data availability, the large and growing spread in prices between private and public payers implies hospitals are likely sensitive to opportunities to maximize reimbursement for these particularly high-priced patients.

We assessed whether the share of admissions for privately insured patients at hospitals is associated with the number of diagnoses filed on Medicaid patients’ claims in New York State (NYS). Because Medicaid payment rates are lower relative to private payments across hospitals,^3,10,11^ increases in the thoroughness of diagnostic coding for these patients likely reflect spillovers from institutional-level administrative investments at sites that see high numbers of privately insured patients. We also examined whether additional diagnoses were disproportionately likely to be those that have been shown to be more intensely coded in response to payment incentives or are easily upcoded.^12^

## Methods

### Data and Study Sample

This cross-sectional study leveraged NYS Medicaid fee-for-service and managed care claims from 2010 through 2017 to identify Medicaid enrollees with multiple inpatient visits. Publicly available all-payer hospitalization data from the Statewide Planning and Research Cooperative System (SPARCS) was used to calculate the share of patients insured by private payers, Medicare, Medicaid, and other payers each year for each hospital. Medicare Advantage and Medicaid Managed Care plans are categorized as public payers in SPARCS.

For the main analyses, we included all NYS Medicaid enrollees with 2 or more hospitalizations between 2010 and 2014 in at least 2 different facilities (359 311 enrollees). Newborn stays were excluded and delivery stays for the birthing parent were included. Patients dually enrolled in Medicare were included because dual-eligible patients face inpatient copayments that are covered by Medicaid, giving us access to their inpatient claims. Only patients admitted to general acute hospitals were included; those at inpatient substance use disorder facilities, mental health hospitals, postacute rehabilitation hospitals, or birthing-only facilities were excluded. Admissions at facilities with fewer than 1000 annual admissions across payers were excluded. We manually linked hospitals in the two data sets using hospital zip code and name to assign the payer mix to facilities in the Medicaid data. All 153 SPARCS facility IDs were successfully linked to Medicaid IDs.

Patient demographics were collected by self-report during Medicaid enrollment. “Other” race refers to non-Hispanic enrollees who identify as a race not listed on the enrollment form or who are American Indian or Alaska Native (the numbers for this group were too low to report separately).

The New York University institutional review board approved the study and waived the need for informed consent because the research only involved secondary analyses of deidentified administrative data and was deemed to pose minimal risk to participants. This study followed the Strengthening the Reporting of Observational Studies in Epidemiology (STROBE) reporting guidelines. Analyses were conducted from 2019 to 2021.

### Outcomes

Our outcome was the number of unique *International Classification of Diseases, Ninth and Tenth Revisions, Clinical Modification *(*ICD-9* or *ICD-10*) diagnoses on the Medicaid claim at discharge. We Winsorized the number of diagnoses at the 99th percentile (25 diagnoses) to minimize potential bias from outliers.^13^

Secondary outcomes were the probability that at least 1 of the nonprimary diagnoses on the claim was from a list of “commonly upcoded” conditions that were identified in prior literature as more intensely coded in response to payment incentives (depression, neuropathy, chronic obstructive pulmonary disease, nephritis, chronic kidney disease, kidney failure, and diabetes complications) and the probability that at least 1 of the nonprimary diagnoses was from a list of conditions unlikely to be upcoded (epilepsy, multiple sclerosis, cystic fibrosis, schizophrenia, paralysis, skin ulcers, septicemia, and skull fractures).^12^ Although the list of diagnoses susceptible to upcoding were identified based on Medicare Advantage incentives, they represent conditions that have been viewed in the literature as more “discretionary” because they typically do not involve labratory or radiological tests or are sequelae of underlying conditions.

In secondary analyses, we examined within-patient moves across hospitals in different quartiles of the share of privately insured patients (ie, “switchers”). The outcome was the net change in the diagnosis count from one admission to the next.

### Statistical Analysis and Estimation

We estimated a discharge-level individual fixed effects model using multivariable linear regression, to obtain the within-patient association of payer mix and diagnosis count. Payer mix was measured as the proportion of privately insured patients at the admitting hospital in each year. Patient fixed effects were used to account for time-invariant patient-specific factors, and calendar year fixed effects accounted for overall trends. Time-varying patient characteristics were used as covariates, including number of procedures (measured as the number of unique *ICD-9* inpatient procedures), the Clinical Classification Software (CCS) category for the primary diagnosis, dual Medicaid-Medicare enrollment, and age at admission. We clustered standard errors at the hospital-year level.^14^

To measure the association between payer mix and the probability of having secondary diagnoses from the list of conditions susceptible to upcoding, we used the same individual fixed-effects specification as described, modeling the outcome in a linear probability model.

### Sensitivity Analyses

We conducted several sensitivity analyses. Despite the use of individual fixed effects and time-varying covariates, our results may be biased by sicker patients selecting hospitals with high shares of privately insured patients (because of perceived higher-quality care, for example). We therefore limited analyses to high-acuity, emergency conditions for which patients are less likely to select their hospital in advance (ie, stroke, acute myocardial infarction, and patients arriving via ambulance). We also conducted sensitivity analyses excluding NYC public hospitals (“Health+Hospitals” [HH] facilities)—a large public system that has acknowledged persistent under-coding in the past^15^—to test whether associations were robust to the absence of this influential public hospital system. We also conducted analyses removing procedure count as a covariate because the number of procedures may itself be a function of payer mix; including distance to the hospital as a covariate; and including an indicator for having a major operation during the admission as a covariate.

Although fewer years of data were available, we assessed whether patterns persisted following the transition to *ICD-10* coding (effective October 2015) by replicating our analysis with data from 2016 to 2017. We analyze these patients separately from the 2010 to 2014 sample because *ICD-10* greatly increased the number of codes available and represented a major shock to coding practices nationwide. Patients in the 2016 to 2017 replication were required to have 2 or more admissions in at least 2 different facilities in the 2-year period.

### Secondary Analysis

In a secondary analysis, we conducted a patient-level analysis to test whether the difference in the number of diagnoses is associated with the difference in the share of privately insured patients between the first and second hospital. Relative to the main model, this analysis accounts for the order in which each patient visited the facilities and for natural increases in comorbidities over time owing to disease progression. This model was similar to the analysis of Song et al^9^ of changes in diagnoses among Medicare enrollees moving regions.

The outcome was the difference in the number of diagnoses between the first and second hospitalization. We categorized each hospital into quartiles based on the share of privately insured patients, with the 4th quartile denoting the highest share of privately insured patients. The explanatory variable was a 16-level variable indicating each combination of the first and second hospital’s quartile (eg, Q1 to Q4, Q1 to Q1). We estimated a linear model, controlling for patient characteristics and year fixed effects. We clustered standard errors at the hospital-year level.

## Results

### Sample Characteristics

Our data contained 1 614 630 admissions among 359 311 enrollees. Table 1 shows descriptive statistics for the sample. Overall, 153 facilities were included in the analysis. The mean annual share of privately insured patients at these facilities was 24.6% (10th percentile: 9.8%, 90th percentile: 40.6%); the mean annual share insured by Medicaid was 27.4% (10th percentile: 10.3%, 90th percentile: 52.8%). The share of dual-eligible enrollees, share of children, and racial and ethnic breakdown of Medicaid patients varied substantially by quartile of share of privately insured patients. All HH facilities fell in the lowest quartile.

**Table 1.** Descriptive Statistics of Sample

Variable	All admissions	1st Quartile^a^ (lowest share private)	2nd Quartile	3rd Quartile	4th Quartile (highest share private)
Age, mean (SD), y	48.2 (20.1)	48.1 (19.3)	50.6 (20.5)	47.6 (20.1)	47.1 (21.2)
Younger than 18 y	5.6	4.8	4.3	5.4	8.1
Dual eligible	26.3	22.1	31.2	28.2	28.2
Sex (%)					
Female	51.4	47.4	54.5	54.1	53.5
Male	48.6	52.6	45.5	45.9	46.5
Race and ethnicity^b^ (%)					
Asian	4.6	4.5	4.9	4.0	5.5
Black	28.6	36.3	23.9	22.5	24.7
Hispanic	23.3	27.8	23.6	18.8	19.2
White	30.1	16.8	35.1	42.8	37.6
Unknown	8.0	8.1	7.3	8.1	8.3
Other	5.4	6.6	5.2	3.9	4.7
Primary CCS^c^ (%)					
Alcohol use disorder	7.3	8.9	5.7	7.1	5.7
Substance use disorder	5.5	5.7	6.3	5.8	4.2
Schizophrenia	3.5	6.6	1.8	1.4	1.2
Septicemia	3.4	2.8	4.7	3.5	3.3
Mood disorders	3.2	4.9	2.7	2.3	1.7
Diabetes complications	2.9	3.0	3.1	2.9	2.5
Asthma	2.8	3.6	2.7	2.0	2.2
Congestive heart failure	2.6	2.6	2.9	2.3	2.4
Chest pain	2.5	2.9	2.5	2.3	2.2
Pneumonia	2.2	2.0	2.5	2.3	2.2
Other	64.1	57.0	65.1	68.1	72.4
Diagnoses, mean (SD)	9.6 (5.5)	8.6 (4.9)	10.0 (5.7)	10.2 (5.6)	10.5 (5.8)
Procedures, mean (SD)	2.2 (2.4)	2.1 (2.3)	2.1 (2.3)	2.2 (2.5)	2.6 (2.7)
NYC Public hospital (HH)	18.7	47.5	0	0	0
Admissions, no	1 614 630	635 754	285 064	351 512	342 300

Abbreviations: CCS, Clinical Classification Software categories; HH, NYC Health+Hospitals (public hospital system).

^a^ Quartiles are calculated using the share of privately insured patients in each year for the 153 facilities.

^b^ All racial and ethnic categories are non-Hispanic, except the Hispanic category. “Other” includes enrollees who identify as a race or ethnicity not listed on the enrollment form or as American Indian or Alaska Native.

^c^ Only the top 10 CCS categories are shown (top 10 were selected based on the entire sample).

### Model Results

Results from the individual fixed effects model showed that a 1 percentage point (pp) increase in the share of privately insured patients at a hospital was associated with an increase of 0.03 diagnoses coded (95% CI, 0.03-0.04; *P* < .001) (Table 2). Put differently, 1 additional diagnosis on average was recorded when a Medicaid-insured patient was seen in a hospital with 40% privately insured patients (90th percentile) compared with when that same patient was seen in a hospital with just 10% privately insured patients (10th percentile) (95% CI, 0.81-1.26 additional diagnoses).

**Table 2.** Regression Results, Including Subgroup Analyses and Sensitivity Checks

Change in diagnosis count, SE			
**For each pp increase in share privately insured**			
Model 1: individual FEs	All admissions^a^	Stroke/AMI only	Ambulance only
Share privately insured	0.034^b^ (0.004)	0.059^b^ (0.006)	0.045^b^ (0.004)
Admissions, no.	1 614 630	12 721	227 608
**When switching** ^c^ ** quartiles for sequential admissions**			
Model 2	All admissions	Stroke/AMI only	Ambulance only
Q1 to Q4 (vs Q1 to Q1)	1.37^b^ (0.08)	2.18^b^ (0.62)	1.45^b^ (0.18)
Q4 to Q1 (vs Q1 to Q1)	–1.67^b^ (0.09)	–3.49^b^ (0.62)	–2.27^b^ (0.19)
Pairs of admissions, no.	675 216	1617	52 867

Abbreviations, AMI, acute myocardial infarction; FE, fixed effects model; pp, percentage point; SE, standard error.

^a^ Individual FE indicates the individual fixed effects models, which are adjusted for the identification of each patient to isolate the within-patient association between payer mix and diagnosis count.

^b^ *P* < .001.

^c^ Switching indicates the switchers model, in which pairs of subsequent admissions for the same patient were used to flexibly model the change in diagnosis counts between each quartile of hospital, as measured by share of privately insured patients.

In the secondary analysis, we found that those who went from a hospital in the 1st quartile of privately insured patients (9% privately insured on average) to one in the 4th quartile (41% privately insured on average) received 1.37 additional diagnoses (95% CI, 1.21-1.53; *P* < .001) relative to those whose admissions were both in Q1 hospitals. In contrast, patients going from a 4th quartile hospital to one in the 1st quartile had 1.67 fewer diagnoses (95% CI, −1.84 to −1.50; *P* < .001). The mean changes in diagnosis count when switching between each of the quartiles are shown in the Figure (15 types of switches, using a Q1-to-Q1 switch as the reference). This model used the subset of 675 216 pairs of admissions in which an admission was to a different facility than that of the preceding admission (ie, sequential admissions to the same hospital were excluded).

**Figure.**
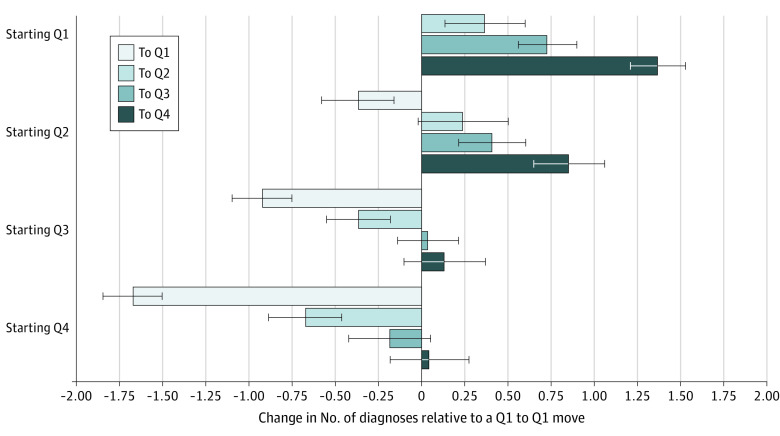
Adjusted Change in Number of Diagnoses When Switching Between Hospitals in Each of the Quartiles of Share of Privately Insured Patients, Relative to a Q1 to Q1 Move Each bar shows the mean adjusted change in the total number of diagnoses recorded on an inpatient claim when a patient switches between 2 different hospitals in subsequent admissions relative to the change among patients making a Q1 to Q1 move. Bars are grouped by the quartile of privately insured patients of the hospital of their initial admission; each bar within the group shows the change in the number of diagnoses as those patients are subsequently admitted to other hospitals, color coded by the quartile of the hospital for their second admission. Q1 are hospitals with the lowest share of privately insured patients; Q4 are the hospitals with the highest share of privately insured patients.

### Characteristics of Nonprimary Diagnoses

Overall, 59% of admissions included at least 1 commonly upcoded nonprimary diagnosis, and 26% of admissions included a nonprimary diagnosis deemed unlikely to be upcoded. In an individual fixed effects linear probability model, nonprimary diagnoses in hospitals with a higher share of privately insured patients were more likely to be from a list of commonly upcoded conditions. Each pp increase in private patient share was associated with a 0.08 pp increase in the probability of receiving at least 1 “commonly upcoded” nonprimary diagnosis for the same Medicaid-insured patient (95% CI, 0.06 to 0.10; *P* < .001). Put differently, the probability of receiving a commonly-upcoded supplemental diagnosis increased by 2.50 pps when a Medicaid-insured patient was seen in a hospital with 40% privately insured patients compared with when they were seen in a hospital with just 10% privately insured patients (95% CI, 1.93-3.07). In contrast, no such relationship existed for diagnoses that were unlikely to be upcoded per prior literature (*b* = 0.00; 95% CI, −0.01 to 0.02; *P* = .54).

### Sensitivity and Subgroup Analyses

Our results persisted in subgroups who were less likely to self-select into more well-resourced hospitals based on comorbidity complexity because these subgroups were admitted for nonscheduled, nonelective acute conditions and would likely go to the nearest site. In a subanalysis of 12 721 admissions with a primary diagnosis of acute stroke or acute myocardial infarction (AMI), we found an association of slightly larger magnitude than the main finding (0.06 additional diagnoses per 1 pp increase in share privately insured; 95% CI, 0.05-0.07; *P* < .001). Similarly, our results persisted among the 227 608 admissions that arrived via ambulance (0.04 additional diagnoses per 1 pp increase in share privately insured; 95% CI, 0.04-0.05; *P* < .001). Table 2 compares these results to the main analysis. The change in the number of diagnoses from one admission to the next were also larger for these subgroups when analyzed in the switchers model (Table 2). eTables 1 and 2 in the Supplement show that we obtained similar results from additional robustness checks: excluding HH facilities, removing procedure count as a covariate, and adding distance to the hospital and presence of a major operation as covariates.

Our results persisted in a replication using 2016 to 2017 data, representing an era wholly after *ICD-10* implementation. The enrollees in this replication differed from the main analysis (because they had 2 admissions in just 2 years, rather than 5 years), but we found a similarly large association: each pp increase in private share was associated with 0.06 additional diagnoses (95% CI, 0.05-0.07; *P* < .001). Using the switchers specification, patients who went from a hospital in the 1st quartile of privately insured patients to a hospital in the 4th quartile received 1.85 additional diagnoses (95% CI, 1.51-2.19; *P* < .001) relative to those whose admissions were both in Q1 hospitals.

## Discussion

The extent to which insurance mix might influence administrative billing practices and investments is not understood. Prior work suggests that hospitals often recognize and respond to opportunities to capitalize on higher prices from payers by increasing thoroughness of coding, such as increases in coding intensity observed when prices for certain Medicare admissions are raised.^7,8,16^ Other literature has documented^17,18,19^ spillovers of responses to payment incentives from 1 payer onto other payers, such as in variation in managed care share. Given this context, we tested whether institutional-level payer mix was associated with the number of diagnoses on Medicaid patients’ inpatient claims.

We found that a Medicaid patient moving from a hospital in the lowest quartile of share of privately insured patients to a hospital in the top quartile will have 1 to 2 additional diagnoses recorded on their claim, on average. This association is unlikely to be owing to selection of more complex patients into well-resourced hospitals because the association persisted for patients admitted for urgent conditions such as AMI and stroke, and for those arriving via ambulance. The probability of receiving a secondary diagnosis from a list of conditions susceptible to upcoding—such as neuropathy and depression—increased with a hospital’s share of privately insured patients.

Because our sample was composed entirely of Medicaid patients for whom prices are low, the observed effects may represent spillovers of administrative practices and investments made by hospitals that are responding to incentives stemming from private prices. Hospitals that see a larger number of privately insured patients stand to benefit more from better administrative and coding infrastructure because each patient (on average) represents a higher potential reimbursement. Although it is possible that revenue-minded facilities seek out privately insured patients and choose to invest in intensive coding as 2 independent actions, our finding that payer mix is meaningfully associated with diagnostic coding warrants further attention into how hospitals respond to the opportunity for high reimbursements from some payers.

Taken in the context of the broader literature on coding behavior, our findings point to insurance mix of hospitals as a specific and important consideration for researchers and policy makers relying on claims data. Future research should aim to document downstream events associated with this variation in coding behavior, such as longer-term patient outcomes. Changes in coding behavior owing to payment incentives have been associated with patient survival in the context of heart disease, highlighting the potential for health effects.^16^ Risk-adjusted measures of mortality and quality rely on diagnostic coding, which can greatly affect a hospital’s reputation.^20^ Future work could consider causal approaches that can strengthen our understanding of whether hospitals respond to price incentives with specific administrative investments. Hospital billing and coding behavior are under increased scrutiny as inpatient expenditures grow.^21,22^ Hospitals’ behavioral responses to higher private prices may affect patients of other payers such as Medicaid, suggesting that this policy problem may be far-reaching.

### Limitations

This study has limitations. First, our main analysis only examined claims through 2014 because the transition to *ICD-10* in 2015 resulted in incomparability across periods and served as its own shock to hospitals. However, we replicated our results using data from the post–*ICD-10* era (2016-2017) and obtained similar estimates. Second, despite our use of individual-level fixed effects and ability to leverage patients who switched types of hospitals between sequential admissions, we cannot be sure that patients are not selecting into well-resourced hospitals when their condition is more serious or complex. Although we address this possibility by conducting subanalyses of admissions for urgent conditions and find consistent results, unobserved patient selection into sites may still drive a portion of the association. Our study relies on patients with at least 2 admissions to 2 different hospitals in a 5-year period (and over just 2 years for the *ICD-10* replication); this group of patients was likely sicker than the average Medicaid patient, limiting generalizability. Still, this population is likely also the most costly and consequential, suggesting that these findings are likely still important for local Medicaid programs and researchers who rely on claims-based diagnoses for risk adjustment, rate-setting, or surveillance.

## Conclusions

Patient diagnoses are used for a range of administrative, research, and policy purposes, including risk-adjustment, rate setting, quality measurement, and disease surveillance, yet the findings of this cross-sectional study suggest that variation in coding intensity may not reflect true underlying variation in patient health. Our finding that a hospital’s share of privately insured patients is a powerful predictor of coding intensity is consistent with prior research that shows that the number of diagnoses recorded on patient claims is related to the average local practice intensity.^9^ Hospitals serving high shares of privately insured patients have more resources and more incentive to opt for costly coding infrastructure and to hire and train administrative teams who can use such infrastructure.^6^ To the extent that diagnoses drive reimbursement and quality scores, this may create a feedback loop that further benefits highly-reimbursed facilities and exacerbates inequity in resources.

## Notes

Supplement. eTable 1. Robustness checks (excluding HH facilities and removing procedure count) eTable 2. Additional covariate checks in the primary specification (adding distance to hospital and presence of major operation)
