# Supplementary material for: Association of Insurance Mix and Diagnostic Coding Practices in New York State Hospitals
Source: JAMA Health Forum. 2022 Sep 2;3(9):e222919. doi: 10.1001/jamahealthforum.2022.2919 (PMC9440394; doi:10.1001/jamahealthforum.2022.2919)
Supplement: Supplement. — eTable 1. Robustness checks (excluding HH facilities and removing procedure count) eTable 2. Additional covariate checks in the primary specification (adding distance to hospital and presence of major operation) [file jamahealthforum-e222919-s001.pdf]

Supplemental Online Content

Dragan KL, Desai SM, Billings J, Glied SA. Association of insurance mix and diagnostic coding practices in New York state hospitals. *JAMA Health Forum*. 2022;3(9):e222919. doi:10.1001/jamahealthforum.2022.2919

eTable 1. Robustness checks (excluding HH facilities and removing procedure count)

eTable 2. Additional covariate checks in the primary specification (adding distance to hospital and presence of major operation)

This supplemental material has been provided by the authors to give readers additional information about their work.

© 2022 Dragan KL, et al. *JAMA Health Forum*.

eTable 1. Robustness checks (excluding HH facilities and removing procedure count)

Panel A: Change in diagnosis count for each pct point increase in share privately-insured

	Excluding HH *Individual FEs*[a] *All admissions*		***Removing Procedure Count Covariate*** *Individual FEs* *All admissions*	
Share privately-insured	0.033*** (0.004)		0.043*** (0.003)	
N (admissions)	1,312,346		1,614,630	

Panel B: Change in diagnosis count when switching quartiles for sequential admissions

	Switchers[c] *All admissions*		*Switchers* *All admissions*	
Q1 to Q4 (vs. Q1 to Q1)	1.34*** (0.13)		1.61*** (0.08)	
Q4 to Q1 (vs. Q1 to Q1)	-1.63*** (0.13)		-1.62*** (0.09)	
N (pairs of admissions)	438,200		675,216	

[a] HH, NYC Health + Hospitals (public hospital system)
[b] "Individual FE" indicates the individual fixed effects model, which are adjusted for the ID of each patient to isolate the within-patient association between payer mix and diagnosis count.
[c] "Switchers" indicates the switchers model, in which pairs of subsequent admissions for the same patient are used to flexibly model the change in diagnosis counts between each quartile of hospital, as measured by share of privately-insured patients.
***p<0.0001

© 2022 Dragan KL, et al. *JAMA Health Forum*.

eTable 2: Additional covariate checks in the primary specification (adding distance to hospital and presence of major operation)				
	Controlling for distance to hospital<br>*Individual FEs[a]*<br>*All admissions*		***Controlling for presence of major operation***<br>*Individual FEs*<br>*All admissions*	
Share privately-insured	0.035***<br>(0.006)		0.035***<br>(0.006)	
N (admissions)	1,420,560		1,614,630	
[a] HH, NYC Health + Hospitals (public hospital system)<br>[b] "Individual FE" indicates the individual fixed effects model, which are adjusted for the ID of each patient to isolate the within-patient association between payer mix and diagnosis count.<br>***p<0.0001				

© 2022 Dragan KL, et al. *JAMA Health Forum*.
